# Treatment of gram-positive deep sternal wound infections in cardiac surgery -experiences with daptomycin-

**DOI:** 10.1186/1749-8090-6-112

**Published:** 2011-09-19

**Authors:** Aron F Popov, Jan D Schmitto, Ahmad F Jebran, Christian Bireta, Martin Friedrich, Direndra Rajaruthnam, Kasim O Coskun, Anselm Braeuer, Jose Hinz, Theodor Tirilomis, Friedrich A Schoendube

**Affiliations:** 1 Department of Thoracic Cardiovascular Surgery, University of Göttingen, Germany; 2 Department of Cardiothoracic Transplantation and Mechanical Circulatory Support, Royal Brompton & Harefield Hospital, London, UK; 3 Division of Cardiac Surgery, Department of Surgery, Brigham and Women's Hospital, Harvard Medical School, Boston, MA, USA; 4 Department of Anaesthesiology, Emergency and Intensive Care Medicine, University of Göttingen, Germany

**Keywords:** Cardiac surgery, Sternal infection, Antibiotic therapy, Daptomycin

## Abstract

The reported incidence of deep sternal wound infection (DSWI) after cardiac surgery is 0.4-5% with *Staphylococcus aureus *being the most common pathogen isolated from infected wound sternotomies and bacteraemic blood cultures. This infection is associated with a higher morbidity and mortality than other known aetiologies. Little is reported about the optimal antibiotic management. The aim of the study is to quantify the application of daptomycin treatment of DSWI due to gram-positive organisms post cardiac surgery.

We performed an observational analysis in 23 cases of post sternotomy DSWI with gram-positive organisms February 2009 and September 2010. When the wound appeared viable and the microbiological cultures were negative, the technique of chest closure was individualised to the patient.

The incidence of DSWI was 1.46%. The mean dose of daptomycin application was 4.4 ± 0.9 mg/kg/d and the average duration of the daptomycin application was 14.47 ± 7.33 days. In 89% of the patients VAC therapy was used. The duration from daptomycin application to sternal closure was 18 ± 13.9 days. The parameters of infection including, fibrinogen (p = 0.03), white blood cell count (p = 0.001) and C-reactive protein (p = 0.0001) were significantly reduced after daptomycin application. We had no mortality and wound healing was successfully achieved in all patients.

Treatment of DSWI due to gram-positive organisms with a daptomycin-containing antibiotic regimen is safe, effective and promotes immediate improvement of local wound conditions.

Based on these observations, daptomycin may offer a new treatment option for expediting surgical management of DSWI after cardiac surgery.

## Introduction

Deep sternal wound infection (DSWI) is a rare complication after median sternotomy. The reported incidence varies from 0.4%-5%, and *Staphylococcus aureus *(gram-positve organism) is the most common pathogen isolated from infected sternal wounds and even in blood cultures in these patients [1,2]. This complication is often associated with significant morbidity, including prolonged hospitalization, additional surgical procedures together with expensive antibiotic therapy and mortality rates of up to 45% [2-4]. Mediastinitis is usually classified into five types based on the time of first presentation, the existence or absence of risk factors and the presence or absence of single or multiple failed therapeutic trials (El Oakley and Wright) [5]. The management of mediastinitis involves many procedures and the choice of the surgical strategy is usually based on the El Oakley and Wright classification. A wide range of strategies have been proposed for the treatment of DSWI, including an intense course of directed antibiotic therapy together with a series of debridements and multiple dressing changes. Closed irrigation may be used, but eventually reconstruction with vascularised soft tissue or muscle flaps can be necessary [6].

Nonetheless, despite the use of perioperative antibiotic prophylaxis, modern surgical techniques and careful wound treatment, DSWI will likely to remain a complication of median sternotomy. As we see an increase in the comorbidities identified as risk factors for DSWI namely diabetes and obesity, refining the therapeutic options to mediastinitis becomes even more important [7].

Appropriate medical treatment of *Staphylococcus aureus *induced of DSWI very often involves the systemic administration of vancomycin. However, the use of this agent has been associated with suboptimal outcomes and can increase the risk of renal failure and the risk developing a drug resistant organism. Because of these deleterious outcomes, there is a definite need to find alternate strategies for patients with mediastinitis post cardiac surgery [8,9].

Daptomycin is a lipopeptide antibiotic approved by the U.S. Food and Drug Administration (FDA) at a dose of 4 mg/kg for the treatment of complicated skin and skin structure infections (cSSSIs) caused by susceptible isolates of certain gram-positive organisms. Daptomycin is bactericidal, and its mechanism of action is by depolarization of the cell membrane [10].

The difference between daptomycin and standard therapy in the treatment of *Staphylococcus aureus methicillin susceptible *(MSSA) infections was up until now not statistically significant, however daptomycin has already been proven to be effective in the treatment of bacteremia and endocarditis caused by MRSA and several case reports exists, documenting its effectiveness in the field of cardiac surgery [11-15]. However, data on the optimal antibiotic management or duration of therapy for DSWI is scarce.

The aim of the study is therefore to describe the application and efficacy of daptomycin in the treatment of DSWI due to gram-positve organisms after cardiac surgery.

## Materials and methods

### Study Population

The following protocol was approved by the local ethics committee of the Medical Faculty, University of Göttingen, Germany. The study was designed as a prospective observational study with a cohort of patients with DSWI following cardiac surgery. After appropriate experience was acquired with the application of daptomycin as an antibiotic therapy in our division, we conducted this prospective study from February 2009 until September 2010. A total of 23 consecutive patients with post-sternotomy mediastinitis from gram-positive organisms (out of 1574 primary sternotomies) were identified, and treated with intravenous daptomycin. All patients had open-heart operations with midline sternotomy in our institution. Patients with sterile dehiscence or superficial sternal wound infections were excluded.

Various preoperative, intra- and postoperative variables were observed and documented consecutively. The patient characteristics included age, gender, body mass index (BMI), class of angina, presence of endocarditis, presence of atrial fibrillation, hypertension, peripheral vascular disease, history of cerebrovascular accident, hypercholesterolemia, history of diabetes, obesity, renal dysfunction, hemodialysis and chronic obstructive pulmonary disease. In addition, preoperative cardiac history and medications were recorded (Table 1).

**Table 1:** Patient and disease characteristics

Variable	n = 23 (%)
Age at operation (years)	71.04 ± 10.77
Male	17 (74)
BMI (kg/m^2^)	24 ± 5
***Risk factors***	
Angina class 4	5 (21)
Active endocarditis	1 (4)
Atrial fibrillation	3 (12)
Hypertension	20 (80)
Peripheral vascular disease	4 (16)
History of CVA	4 (16)
Hypercholesterolemia	10 (40)
Diabetes mellitus	7 (28)
Obesity	4 (16)
Renal dysfunction	10 (40)
Hemodialysis	2 (8)
COPD	6 (24)
***Cardial history***	
CAD	10 (40)
Aortic valve disease	4 (16)
Mitral valve disease	3 (12)
Ejection fraction (%)	47.87 ± 11.10
NYHA class	3 ± 0.36
***Preoperative Medication***	
Beta blockers	14 (56)
ACE inhibitors	13 (52)
Ca^2^-Channel blocker	6 (24)
Diuretics	22 (88)
Aspirin	14 (56)
Antiarrhythmics	1 (4)

BMI: body mass index, COPD: chronic obstructive pulmonary disease, CVA: cerebrovascular accident, CAD: coronary artery disease, NYHA: New York Heart Association, ACE: angiotensin converting enzyme

Perioperative patient variables studied included the cardiac surgical procedure, additive Euroscore, operation time, cardiopulmonary bypass time, aortic clamp time, intensive care unit stay, duration of ventilation, hospital stay, and mortality. Mortality was defined as death occurring within 30 days of the last surgery, regardless of whether the patient was an in-patient or was discharged from the hospital at the time of occurrence.

The postoperative details recorded the quantity of red blood cells suspension and fresh frozen plasma transfused (Table 2).

**Table 2:** Operative and postoperative details

Variable	n = 23	Percentage [%] or range
CABG	13	52
Bilateral internal mammary artery	2	8
AVR	1	4
CABG + AVR	6	24
MVR	1	4
AAR	2	8
Euroscore additive	6 ± 3	
Operation time (min)	273 ± 72	180-420
CPB(min)	134 ± 31	70-245
Aortic clamp time (min)	84 ± 28	49-142
ICU (d)	8.51 ± 17.07	1-80
Duration of ventilation (h)	73 ± 218	5-994
Red blood cells transfused (ml)	1151.77 ± 747.70	0-9067
Fresh frozen Plasma (ml)	243.63 ± 82.21	0-2860
LOS (d)	31.34 ± 33.07	9-140
Survival (%)	100	

CABG: coronary artery bypass grafting, AVR: aortic valve replacement, MVR: mitral valve replacement, AAR: aortic ascending replacement, CPB: cardiopulmonary bypass time, ICU: intensive care unit, LOS: length of stay,

### Infection

Infection was defined by means of clinical assessment, laboratory values, and microbiologic analysis. All patients showed DSWI with gram-positive organisms and were classified according to the criteria proposed by El Oakley and Wright.

Furthermore, mediastinal cultures, previous antibiotic therapy, and modalities regarding daptomycin application were studied. A suspicious wound was treated in our department with a standard microbiological protocol including amoxicillin and ciprofloxacin. If we observed a treatment failure and/or the microbiological results showed sensitivity or resistance to other antibiotics, we changed the antibiotic therapy according the microbiological results. The details are summarised in Table 3.

**Table 3:** Infection Parameter

Variable	n = 23 (%)	Percentage [%] or range
***El Oakly-Wright Score***		
Type I	3	13
Type II	6	26
Type IIIa	1	4
Type IIIb	7	30
Type IVa	2	8
Type IVb	0	
Type V	4	16
***Mediastinal cultures***		
Staph. aureus	11	
MRSA	6	
MRSE	6	
Additional Enterococcus faecium	4	
Duration from operation to culture (d)	34 ± 37	5-155
***Previous antibiotic therapy***	17	
0	6	
1	5	
2-3	7	
4-6	5	
***Daptomycin application ***		
Daptomycin-Application (mg)	4.4 ± 0.9	4-6
Duration (d)	14.47 ± 7.33	9-43
Vacuum therapy	19 (83)	83
Omentumplastic	3	13
Duration from infection to sternal closure (d)	22 ± 13.4	8-58
Duration from Daptomycin application to sternal closure (d)	18 ± 13.9	8-55

MRSA: methicillin resistant S. aureus, MRSE: methicillin resistant S. epidermidis,

### Laboratorial data

Blood tests included fibrinogen, hemoglobin, hematocrit, thrombocytes, white blood cell count (wbc), creatinine, total bilirubin, serum glutamic oxaloacetic transaminase (SGOT), serum glutamic pyruvic transaminase (SGPT), gamma-glutamyltransferase (GGT), creatine phosphokinase (CK), creatine phosphokinase-MB (CK-MB), C - reactive protein (CRP), and lactate dehydrogenase (LDH). Blood tests were done prior to commencing treatment with daptomycin, then alternate days thereafter, upon discontinuing this therapy, when patients were discharged to rehabilitative care (Table 4).

**Table 4:** Laboratorial data

Variable	Reference	Before Daptomycin	After Daptomycin	P-value
Fibrinogen (mg/dl)	170-400	674 ± 109	603 ± 125	0.03
Hemoglobin (g/dl)	11.5-15.0	10.4 ± 1.6	9.4 ± 1.3	0.008
Hematocrit (%)	35-46	32 ± 4.8	29 ± 3.1	0.005
Thrombocyte (x 10^3^/μl)	150-350	392 ± 164	334 ± 94	0.21
WBC (x 10^3^/μl)	4.0-11.0	12 ± 4.2	9 ± 3.2	0.001
Creatinine (mg/dl)	0.55-1.02	1.17 ± 0.58	1.12 ± 0.53	0.69
Total Bilirubin (mg/dl)	≤ 1.2	0.44 ± 0.21	0.40 ± 0.25	0.53
SGOT (U/I)	≤ 31	23 ± 16	32 ± 46	0.21
GPT (U/I)	≤ 34	23 ± 13	50 ± 92	0.24
GGT (U/I)	≤ 38	88 ± 61	94 ± 108	0.77
CPK (U/I)	≤ 170	51 ± 37	50 ± 44	0.92
CK-MB (U/I)	≤ 17	23 ± 29	14 ± 7	0.19
CRP (mg/l)	≤ 8.0	118 ± 72	35 ± 32	0.0001
LDH	≤ 232	246 ± 71	212 ± 55	0.05

WBC: white blood cell count

SGOT: serum glutamic oxaloacetic transaminase,

SGPT: serum glutamic pyruvic transaminase

GGT: Gamma-glutamyltransferase

CPK: creatine phosphokinase

CK-MB: creatine phosphokinase-MB

CRP: C-reactive protein

LDH: Lactate dehydrogenase

### Statistics

Continuous variables are presented as mean ± standard deviation, and categorical variables are presented as absolute numbers or percentage. Data were checked for normality before statistical analysis. Comparisons of continuous variables laboratorial data with deep sternal wound infections were made with Student's paired t-test. P < 0.05 was considered statistically significant. All statistical analyses were performed using commercially available software (SPSS for Windows, SPSS Inc. Chicago, IL, USA).

## Results

### Patients' characteristics and perioperative details

Twenty-three patients (6 females and 17 males) were included in the study. Their characteristics are shown in Table 1.

Thirteen patients developed deep sternal wound infection following coronary artery bypass grafting (CABG, including two patients with bilateral internal mammary artery), one patient following aortic valve replacement (AVR), six patients after CABG combined with AVR, one patient following mitral valve replacement (MVR), and two patients following ascending aortic replacement (AAR). The mean operation time was 273 ± 72 min (range, 180 to 240 minutes), the median CPB time at surgery was 134 ± 31 minutes (range, 70 to 245 minutes), and median aortic cross clamp time was 84 ± 28 minutes (range, 49 to 142 minutes). The median length of ICU stay was 8.51 ± 17.07 days (range, 1 to 80 days), median time of ventilation 73 ± 218 hours (range, 5 to 994 hours), and median hospital stay was 31.34 ± 33.07 days (range, 9 to 140 days). Furthermore, the administration of red blood cells was 1151.77 ± 747.70 ml (range, 0 to 9067 ml) and of fresh frozen plasma was 243.63 ± 82.21 ml (range, 0 to 2860 ml). A surveillance of 100% was achieved and wound healing was successfully established in all patients at the time of discharge. All details are summarized in table 2.

### Management of Deep Sternal Wound Infection

All the patients were classified according to the criteria proposed by El Oakley and Wright: type I in three patients, type II in six, type IIIa in one, type IIIb in seven, in type IVa in two, and type V in the remaining four. The patients underwent initial surgical revision, at which time a choice of the most suitable procedure was made. This included surgical wound debridement together with continuous irrigation in some instances.

The decision regarding closure was further based on negative wound cultures and the absence of signs of local and systemic infection. Nineteen (83%) patients underwent vacuum-assisted closure (VAC) therapy. Three (13%) of them with persistent local wound infection underwent an additional Omentumplasty prior to definitive chest closure. Four (17%) patients did not require further intervention after initial debridement and the chest was closed without additional surgical procedures. The median duration from infection to sternal closure was 22 ± 13.4 days (range, 8 to58 days) (Table 3).

### Bacteriologic Findings

The bacteriologic etiology was confirmed with wound culture and the median time interval between the initial cardiac operation with sternotomy and the diagnosis of deep sternal infection in this cohort was 34 ± 37 days (range, 5-155 days). Eleven isolates were *Staphylococcus aureus methicillin susceptible*, six were methilicin-resistant *Staphylococcus aureus *and another six were methicillin resistant *Staphylococcus epidermidis*. There 4 isolates as *Enterococcus faecium *were accompanying. Wound classification and mediastinal cultures of the group are given in Table 3.

### Antibiotic application

Seventeen (74%) patients received a previous antibiotic regimen before administered daptomycin. Of these patients, five had 4-6 antibiotics, seven had 2-3 antibiotics, and five had single antibiotic before daptomycin application. Treatment failure was the reason for changing to daptomycin. The remaining six received daptomycin as a first antibiotic therapy.

The median final dose of daptomycin was 4.4 ± 0.9 mg/kg/d intravenously (range, 4 to 6 mg/kg/d), and the median duration of daptomycin administration was 14.47 ± 7.33 days (range, 9 to 43 days). Furthermore, the median duration from daptomycin application to definitive sternal closure was18 ± 13.9 days (range, 8 to 55 days). There were no adverse events related to the application of daptomycin. Details are summarized in table 3.

### Laboratory data

Compared with the laboratory data before daptomycin application, median fibrinogen, hemoglobin, hematocrit, wbc, and plasma CRP levels declined significantly until discharge (fibrinogen: 674 ± 109 mg/dl and 603 ± 125 mg/dl, respectively, p = 0.03; hemoglobin: 10.4 ± 1.6 g/dl and 9.4 ± 1.3 g/dl, respectively, p = 0.008; hematocrit: 32 ± 4.8% and 29 ± 3.1%, respectively, p = 0.005; wbc: 12 ± 4.2 × 10^3^/μl and 9 ± 3.2 × 10^3^/μl, respectively, p = 0.001; CRP: 118 ± 72 mg/l and 35 ± 32 mg/l, respectively, p = 0.0001).

The liver enzymes (SGOT, SGPT, and GGT) levels, thrombocytes, serum creatinine, serum total bilirubin, CPK, CK-MB, and LDH levels remained constant before the first daptomycin application and discharge and did not achieved statistically significance. All laboratory values are shown in table 4.

## Discussion

Over the past three decades, a wide range of strategies have been proposed for the treatment of DSWI. Current forms of treatment for DSWI usually involve a series of debridements, potentially surgical revision with multiple dressing changes, closed irrigation, or reconstruction with vascularized soft tissue or muscle flaps, and an intense course antibiotic treatment [6].

There is no consensus on the optimal management of poststernotomy mediastinitis, but long-term antibiotic treatment is universally accepted as being fundamental to the treatment process [16,17]. Although antimicrobial treatment should be initiated promptly, there is no agreement either on the choice of the most suitable drug or on the preference for a combination therapy over monotherapy, and new antimicrobials are constantly being sought in this era of increasing drug resistance [17]. However, data on the optimal antibiotic regimen of therapy for DSWI is scarce.

Our study reports a single institution's experience in the treatment gram-positive deep sternal wound infections following cardiac surgery. This was the first study to our knowledge that analyzed the application of the new antibiotic, daptomycin in the treatment of DSWI due to gram-positve organisms in cardiac surgery.

Our findings, suggest that treatment of daptomycin-susceptible DSWI with a daptomycin-containing antibiotic regimen is safe, effective in immediately promoting local wound conditions.

*Staphylococcus aureus *is the most common pathogen isolated from sternal wound infections after cardiac surgery and it demonstrates an increasing resistance to wide range of antibiotics [2]. Treatment for *Staphylococcal aureus *DSWI is challenging because of the need for prolonged antibiotic therapy and the risk of haematogenous complications. More importantly, with the incidence of increase of MRSA infection, the accompanying antibiotic therapy has received more attention.

The first in a novel class of cyclic lipopeptide antibiotics daptomycin (Cubicin; Cubist Pharmaceuticals, Inc., Lexington, MA), has already been proven to be effective in the treatment of bacteremia and endocarditis caused by MRSA and several case reports document about its effectiveness in the field of cardiac surgery [11-15]. Furthermore, treatment with daptomycin has also been effective in patients in whom osteomyelitis was diagnosed. Lamp et al. showed that daptomycin had a 94% success rate when used alone in patients with osteomyelitis resulting from infections with gram-positive pathogens including MRSA [18]. Finney et al. reported their experience with daptomycin treatment in patients with osteomyelitis and had a 100% success rate [19]. This observation was consistent with our results. In our cohort we had a 100% success rate with the use of daptomycin. Only a 50% success rate in patients with prosthetic joint infections was reported by Rao and Regalla [20].

It must however be taking in account that in these studies the dose of daptomycin ranges from 4 to 6 mg/kg per day and duration of application varied. In addition, it is very difficult to compare these observations with our results, because our cohort is very small and we have only the observation period up until discharge without long term follow up. Our mean duration of daptomycin-application was 14.47 ± 7.33 days and the mean dose of daptomycin was 4.4 ± 0.9 mg/kg/day. Lamp et al. stated in their study, that daptomycin may produce higher success rates with doses > 4 mg/kg with a long daptomycin therapy (median 35 days) [18]. However, at the time of this study daptomycin 4 mg/kg every 24 hours for cSSSI was the only approved dose and spite of this recommendation we had a 100% success rate in our observations period.

The incidence of spontaneous resistance of daptomycin is until now very low, and there has been no evidence of conjugation-mediated resistance [21].

However, there have been isolated reports of reduced daptomycin susceptibility, but this was not seen in our study [10,22]. We had no mortality and successful wound healing was achieved in all patients. Further, in our study there were no adverse advents. An antibiotic regimen containing daptomycin was generally very well tolerated, and no patient required antibiotic treatment to be discontinued because of daptomycin-related adverse events. This is better than in other studies, which observed adverse event in patients with surgical site infection only or any cSSSI rates of 13% and 18%, respectively [23,24]. Muscle toxicity thought to be related to daptomycin is reported to occur in approximately 3% of patients with complicated skin and skin structure infections (cSSSI), however these observation was not seen in our study [10]. The CPK levels were within in the normal range after commencing treatment with daptomycin.

It is therefore obvious, that daptomycin therapy in combination with the surgical procedures facilitates successful treatment of sternal infections in the majority of the patients. This suggestion is reiterated by the significant decline in the levels of inflammatory markers (fibrinogen, WBC, and CRP) during this combined modality of treatment. Data also suggest that the use of VAC therapy actually shortens the duration of wound healing [25]. The majority (83%) of our patients had accompanying VAC therapy and which may have influenced our high success rate.

In conclusion, our study indicates that the treatment of DSWI of susceptible gram-positive organisms with a daptomycin-containing antibiotic regimen is feasible. The results of this study suggest that daptomycin is efficacious in the treatment of patients with DSWI after cardiac surgery.

However, our study has some limitations. This is an observational analysis with a small sample size; therefore, any conclusions maybe limited in their implications. Further, because of the observational nature of the study, we cannot rule out the presence of other possible confounding variables that might have affected our results. Another limitation is that the results come from a single institution and might not be generalized to other cardiac surgical units. Our study did not evaluate patients preoperatively to identify those who were nasal or planned surgical site carriers of staphylococcal aureus, so as to pre-empt or eradicate these potential pathogens. Moreover, 74% of the patients were pre-treated with other antibiotics. In our point of view it's very difficult starting daptomycin since diagnosis of DSWI, because at this moment we do not have any microbiological results and daptomycin is not a first line antibiotic. A suspicious wound will be treated in our department with a standard microbiological protocol. If we observe a treatment failure and/or the microbiological results shows sensitivity or resistance to other antibiotics, we change the antibiotic therapy according the microbiological results. Furthermore, some of the patients may develop a DSWI after discharge from the hospital and the majority of them are at the postoperative rehabilitation. If a patient develops a DSWI at the rehabilitation, we have no influence on the choice of the antibiotics, because they have their own microbiological protocol. This means, the patient is pre-treated before referral to our department.

Otherwise, this is, to our knowledge, the first study with detailed information on antibiotic treatment with daptomycin for DSWI. However, it is worthy to note that the total number of DSWI were 23 out of 1574 median sternotomies, for the interval from February 2009 up until September 2010. The incidence of sternal infections of 1.46% is at the lower range of reported values in the literature [1]. Data indicate that for heart centers with good surgical practice it is unrealistic to prospectively and monocentrically evaluate the benefit of a specific antibiotic drug compared to standard antibiotic drug protocols. Our approach has demonstrated satisfactory results with regard to the duration and successful management of complex DSWI due to gram-positive organisms. Furthermore, our single centre results suggest that further investigation, for instance in a multicenter trial, is needed to determine the specific role of daptomycin in the treatment DSWI.

## Competing interests

The authors declare that they have no competing interests.

## Authors' contributions

AFP and JDS conceived the study, and participated in its design and coordination. AFP wrote the paper. AFP, AFJ and CB supervised postoperative care and wound management. MF, DR and KOC revised manuscript. TT did data interpretation, AB and JH supervised intraoperative and postoperative anesthesia care and revised manuscript. FAS co-wrote the manuscript and added important comments to the paper. All authors read and approved the final manuscript.
